# *In vitro* exposure of neuronal networks to the 5G-3.5 GHz signal

**DOI:** 10.3389/fpubh.2023.1231360

**Published:** 2023-08-07

**Authors:** Anne Canovi, Rosa Orlacchio, Florence Poulletier de Gannes, Philippe Lévêque, Delia Arnaud-Cormos, Isabelle Lagroye, André Garenne, Yann Percherancier, Noëlle Lewis

**Affiliations:** ^1^Univ. Bordeaux, CNRS, Bordeaux INP, IMS, UMR 5218, Talence, France; ^2^Paris Sciences et Lettres Research University, École Pratique des Hautes Études (EPHE), Paris, France; ^3^Univ. Limoges, CNRS, XLIM, UMR 7252, Limoges, France; ^4^Institut Universitaire de France (IUF), Paris, France

**Keywords:** radio-frequency fields, electrical activity, neuronal networks, *in vitro*, 3.5 GHz, 5G signal

## Abstract

**Introduction:**

The current deployment of the fifth generation (5G) of wireless communications raises new questions about the potential health effects of exposure to radiofrequency (RF) fields. So far, most of the established biological effects of RF have been known to be caused by heating. We previously reported inhibition of the spontaneous electrical activity of neuronal networks in vitro when exposed to 1.8 GHz signals at specific absorption rates (SAR) well above the guidelines. The present study aimed to assess the effects of RF fields at 3.5 GHz, one of the frequencies related to 5G, on neuronal activity in-vitro. Potential differences in the effects elicited by continuous-wave (CW) and 5G-modulated signals were also investigated.

**Methods:**

Spontaneous activity of neuronal cultures from embryonic cortices was recorded using 60-electrode multi-electrode arrays (MEAs) between 17 and 27 days in vitro. The neuronal cultures were subjected to 15 min RF exposures at SAR of 1, 3, and 28 W/kg.

**Results:**

At SAR close to the guidelines (1 and 3 W/kg), we found no conclusive evidence that 3.5 GHz RF exposure impacts the activity of neurons in vitro. On the contrary, CW and 5G-modulated signals elicited a clear decrease in bursting and total firing rates during RF exposure at high SAR levels (28 W/kg). Our experimental findings extend our previous results, showing that RF, at 1.8 to 3.5 GHz, inhibits the electrical activity of neurons in vitro at levels above environmental standards.

## 1. Introduction

For the last two decades, a very large number of research reports has focused on the potential health effects of radiofrequency electromagnetic field (RF-EMF, 100 kHz to 300 GHz) exposures, mainly due to the rise of wireless communication systems. So far, the only characterized effect of RF-EMF on living organisms has been dielectric-relaxation heating. To protect people against adverse effects induced by heating, the International Commission on Non-Ionizing Radiation Protection (ICNIRP) has provided exposure limits for wireless communications devices, generally expressed in terms of specific absorption rate (SAR), in watts per kilogram (W/kg). The recommended limit for whole-body exposure is 0.08 W/kg, while the SAR limit for local exposure of the head is 2 W/kg (1).

Some authors have also reported non-thermal biological effects of low SAR level RF-EMF (i.e., levels below the international guidelines). However, no plausible mechanistic hypotheses have been proposed, making it difficult to draw conclusions based on available experimental results (2–4).

Few epidemiological studies have also suggested an increased risk of glioma associated with extensive mobile-phone use. In 2011, the International Agency for Research on Cancer (IARC) classified RF-EMF as possibly carcinogenic to humans. However, research conducted on animals and cells has failed to confirm the findings of the epidemiological studies, and there is no biophysical mechanism that could explain the carcinogenicity at such low levels of exposure (5).

Nowadays, questions about the potential health effects of RF-EMF exposure remain relevant, given the current deployment of the fifth generation (5G) of wireless networks, designed to improve on the 4G technology with higher data speeds and faster response rates. To avoid network congestion, the 5G technology will use new frequency bands around 3.5 and 26 GHz, in addition to those already deployed for previous technologies like 2G, 3G, and 4G. Due to its short wavelength, the 26 GHz signal cannot penetrate more than 1–2 mm into the body and, therefore, cannot penetrate deeply enough to reach the brain. In contrast, the 3.5 GHz signal can penetrate into the body and reach the cortex (6). Consequently, given the proximity between the mobile phone and the head, the central nervous system (CNS) remains one of the targets of 5G signals at 3.5 GHz.

We previously reported decreased firing and bursting rates of cortical neuronal cultures exposed to a GSM signal at 1.8 GHz for 3 min (7). We then assessed the dose-response relationship and identified potential differences in the response elicited by pulse-modulated GSM and continuous wave (CW) RF fields (7, 8). Both GSM-modulated and CW signals elicited an important decrease in bursting rate during the RF exposure phase at levels higher than environmental guidelines. A recent dosimetry study (9) led us to improve our experimental bench with new custom multi-electrode arrays (MEAs) allowing high exposure homogeneity. Beyond these adjustments, our experiments provided strong evidence of decreased electrical activity of cortical neuronal cultures during RF exposure. In a further effort to identify the inhibitory mechanisms triggered by RF exposure on cortical neurons, we next have shown that they differ from the ones mediated by the activation of GABA A receptors (10).

The research presented here aimed to investigate the potential effects of 3.5 GHz RF exposure on neuronal response *in vitro*, depending on SAR (1, 3, and 28 W/kg) and type of RF signals (CW and 5G). Specifically, we assessed the impact of RF exposure on cortical neuronal networks spiking and bursting activities.

## 2. Materials and methods

### 2.1. Preparation of dissociated cortical neurons

We performed electrophysiological recordings of neuronal networks using MEAs as described previously (10). The active area of each MEA was coated with 0.1 mg/mL poly-L-lysine (PLL) (Sigma-Aldrich) and 0.05 mg/mL laminin (Sigma-Aldrich) for improved adherence.

Dissociated primary cortical neurons were isolated from the cortex of embryonic (E18–E19) Sprague–Dawley rats (Janvier-Labs, Saint-Berthevin, France) as described in Moretti et al. (7). Following 5% isoflurane anesthesia, gestating rats were euthanized by cervical dislocation. Embryos were collected and decapitated. Their cortices were dissected in Dulbecco’s Modified Eagle Medium (DMEM) supplemented with 1% penicillin-streptomycin (Fisher Scientific, Illkirch, France). Cortices were treated for 30 min with an enzymatic solution containing 20 units/mL of papain and 0.005% DNase (Worthington Biochemical Corporation, Lakewood, United States). The fragments were subjected to mechanical dissociation using a 10 mL serological pipette and centrifuged at 300 g for 5 min at room temperature. The supernatant was eliminated, and the pellet composed of cortical cells was placed in a solution containing DNase. This mixture was first placed above an albumin-inhibitor solution to create a discontinuous density gradient and was then centrifuged at 70 g for 6 min at room temperature. Finally, pellet-dissociated cortical cells were suspended in a culture medium composed of neurobasal medium (NBM) (Fisher Scientific) supplemented with 2% B-27, 1% GlutaMAX, and 1% penicillin-streptomycin (Fisher Scientific). Each MEA was plated with 10^5^ cells and kept in a humidified incubator (37°C, 5% CO_2_) until recording. The culture medium was replaced by fresh and pre-warmed medium every 48 h.

### 2.2. Electrophysiological recording system

**Figure 1:**
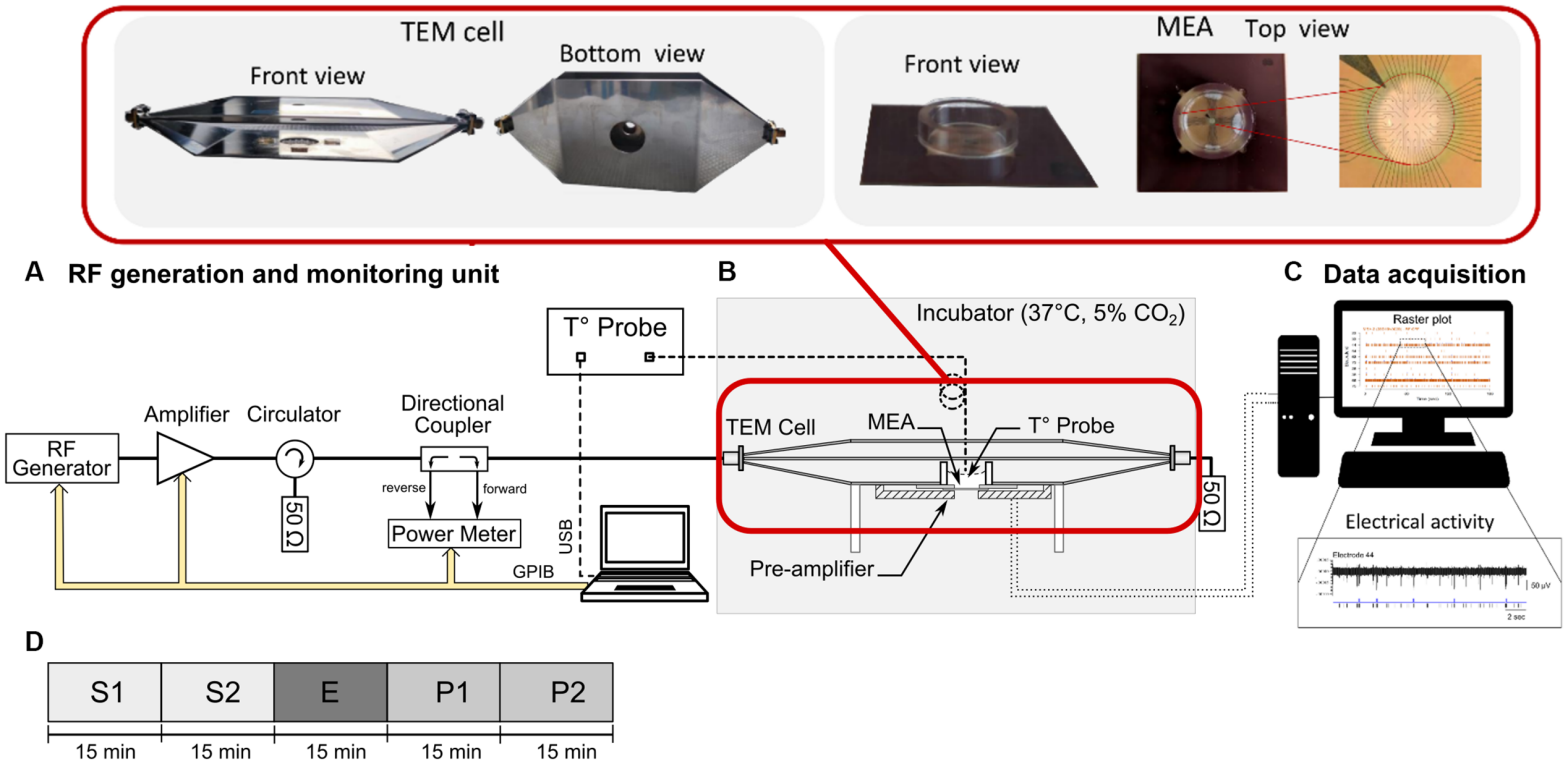
Schematic representation of the experimental setup used for the neuronal cell culture exposure to either continuous wave (CW) or 5G-modulated signal at 3.5 GHz and simultaneous recording of the spontaneous electrical activity of neuronal networks. **(A)** The RF generation and monitoring unit composed of an RF generator, a power amplifier, a circulator, a bidirectional coupler, a power meter, and a computer for the control of the exposure parameters. **(B)** The cell culture incubator containing an open transverse electromagnetic (TEM) cell, docked on a pre-amplifier and connected to the radiofrequency (RF) generation unit and to a 50 Ω load. A microelectrode array (MEA) was placed in between the TEM bottom ground plate and the preamplifier connected to an acquisition unit (MC-card of a desktop computer). A fiber-optic probe was used for the temperature measurements in the culture medium. **(C)** Computer used for the recording and post-processing of the electrical activity of the exposed or sham-exposed neurons. An example of raster-plot and electrical activity recorded on one electrode is also shown. **(D)** Timeline of the exposure protocol divided into 5 phases of 15 min: two pre-exposure phases (S1 and S2), the RF or sham exposure phase (E), and two post-exposure phases (P1 and P2).

In the current study, the electrophysiological interface used to grow neuronal networks was a commercial MEA containing a 60 titanium nitride electrodes array (200 μm spaced with 30 μm-diameter tips) including one internal reference electrode (upper right panel of Figure 1) (60MEA200/30iR-Ti-Upside Down, Multi-Channel Systems, MCS, GmbH, Reutlingen, Germany). The MEA culture chamber was made with a 4.8 mm-high, 19 mm inner-diameter glass ring sealed with biocompatible silicone. This MEA was customized to have the contact pads underneath the printed circuit board (PCB) (9) and connected to the pre-amplifier (MEA1060-Inv, MCS GmbH, gain of 1,200), placed below the MEA, as depicted in Figure 1B. Electrophysiological recordings were performed in a dry incubator to maintain the desired cellular physiological conditions (37°C, 5% CO_2_). To avoid evaporation of the culture medium during the experiment, the MEA was covered with a plastic lid with a removable membrane. Neuronal signals were acquired through an MCS data acquisition board (MC_Card, MCS) at 10 kHz/channel and visualized with the MC Rack software (MCS GmbH) (Figure 1C).

### 2.3. Post-processing of the electrophysiological signal

The electrical activity of neuronal cultures was analyzed using the SPYCODE software (11) developed in MATLAB (The MathWorks, Inc., Natick, MA, United States). Spiking activity was detected using the differential threshold precision timing spike detection (PTSD) method (12). This algorithm detects one spike when the peak-to-peak amplitude of the signal exceeds eight times the standard deviation (SD) of the biological noise in a 2 ms sliding window. The SD of the biological noise was evaluated for each recording channel in the pre-exposure phase. Bursts were then detected using the method described by Pasquale et al. (13). The algorithm is based on the computation of the logarithmic inter-spike interval (ISI) histogram and automatically detects the best ISI threshold for finally distinguishing between spikes inside or outside bursts for each recording channel of the array. An analogue thresholding method was used to detect network bursts, based on an inter-burst interval (IBI) histogram combined with a synchronization criterion implicating at least 20% of the electrodes (13).

### 2.4. RF exposure system

The experimental setup used for RF exposure is described in our previous works (7–10) and schematically shown in Figure 1. Briefly, it consists of three main units including (i) an open transverse electromagnetic (TEM) cell (Figure 1B), (ii) an RF signal generation unit (Figure 1A), and (iii) a computer for monitoring the exposure parameters, namely incident power, and exposure duration (Figure 1A). The incubator shields the system from possible external electromagnetic interferences. The TEM cell length is 160 mm and the widths of the central septum, and the two external plates are 30 mm and 85 mm, respectively. Given the ratio between the TEM cell septum width (30 mm) and total height (21 mm), a cut-off frequency of around 6 GHz is obtained for symmetrical structures. This is high enough to support the TEM cell use up to 3.5 GHz. The specific customization of MEAs was made to ensure highly uniform exposure of the neuronal cultures and to improve the electromagnetic compatibility of the experimental device by allowing the stability of the SAR and temperature during RF exposure (9, 14). During exposure, the MEA was located between the TEM bottom ground plate, through a 24 mm diameter aperture (upper left panel of Figure 1) and pre-amplifier. Moreover, the pre-amplifier was shielded with RF absorbers to avoid residual interference with the recording system. The RF generation unit was located outside the incubator (Figure 1A) and consisted of (i) an RF signal generator (SMBV100A, Rohde & Schwarz) used to generate either CW or 5G-modulated signal at 3.5 GHz, (ii) a 25 dB gain amplifier (Mini-circuits, ZHL-4W-422+, NY, United States), (iii) a circulator (Pasternack, PE83CR1005, CA, United States), (iv) a bidirectional coupler (Mini-circuits, ZGBDC30-372HP+, NY, United States), and (v) a power-meter (N1912A, Keysight, United States) with two power sensors (N1921A, Keysight, United States) to monitor in real-time the incident and reflected powers at the TEM cell input. The 3.5 GHz 5G signal used corresponds to 5G NR (release 15, Digital Standards SMBVB-K444, Rohde & Schwarz) with FDD duplexing, QPSK modulation and 100 MHz channel bandwidth. The signal was led to the TEM cell through a 1.5 m coaxial precision test cable (CBL-1.5 m-SMNM+, Mini-circuits, United States; 1.2 dB insertion loss at 3.5 GHz) and SMA connectors. To consider the losses along the entire chain, the actual power was measured at the end of the coaxial cable, therefore at the TEM cell input. To absorb transmitted power and prevent wave reflection, the TEM cell output port was connected to a 50 Ω load (PE6185, Pasternack, United States), sustaining up to 10 W power with 6 GHz bandwidth.

### 2.5. RF dosimetry

RF dosimetry, i.e., the quantification of the energy absorbed by the biological samples exposed to an electromagnetic field, is fundamental to ensure the reproducibility and reliability of bioelectromagnetic results (15). In this study, well-defined exposure conditions were obtained through numerical and experimental dosimetry. SAR, the electromagnetic power dissipated per unit mass in the exposed sample (W/kg), was quantified as the metric to define the exposure level. Therefore, numerical simulations and temperature measurements were performed to precisely characterize the exposure conditions of this study: 0.7 mL, 2.3 mm height of culture medium exposed at 3.5 GHz.

Numerical dosimetry represents the direct resolution of Maxwell’s equations under the specific exposure conditions performed through computer simulations. As in our previous studies, a custom finite difference time domain (FDTD)-based code was used to extract both local and volume SAR levels of the exposed samples defined as the SAR values averaged over 0.5 mm^3^ and the whole exposed culture medium, respectively (9, 16, 17). All MEA components except the electrodes and the top conductor tracks were simulated. The metallic components were modeled as perfect electric conductors (PEC). Conductivity (*σ*) and relative permittivity (*ε*_r_) used to simulate the culture medium at 3.5 GHz were measured using a slim dielectric probe (85070E Dielectric probe kit, Agilent, United States) and were equal to 3.85 S/m and 70, respectively (at 37°C). The culture chamber, the glass chip, and the PCB were defined as loss-free materials with *ε*_r_ equal to 4.6, 7, and 4.4, respectively. A volume of 0.7 mL of culture medium with a meniscus was used in the simulation. For accurate results, non-uniform adaptive meshing was used with a 100 μm finest meshing size.

**Figure 2:**
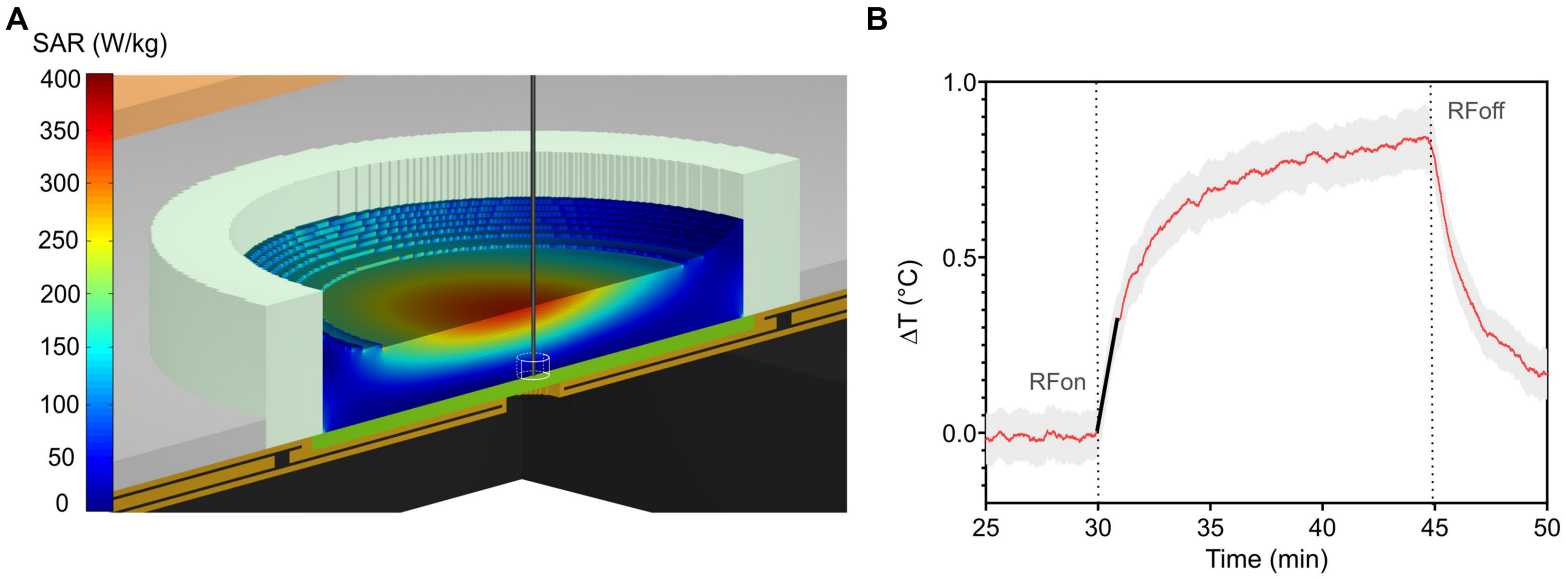
**(A)** Numerical SAR distribution normalized to 1 W incident power at 3.5 GHz along a vertical cut across the TEM cell and MEA filled with 0.7 mL of culture medium. The straight vertical black line represents the thermal probe located at the bottom of the MEA used for the experimental measurement of local SAR within an approximate volume of 0.5 mm^3^. **(B)** Induced-temperature elevation of RF-exposed culture medium with an incident power of 1 W. The red solid trace represents the average temperature elevation, while the grey area represents the standard deviation. The slope at the origin of the transient curve is represented by the solid black line (at *t* = 30 min). Its value is used to determine experimental local SAR.

Besides numerical simulation, the experimental local SAR was extracted from the exponential fitting of the initial phase of the electromagnetic-induced temperature elevation of the culture medium under RF exposure:


(1) $$\mathrm{SAR}=C{\frac{\partial T}{\partial t}|}_{t=t_{0}}$$

where *C* is the sample heat capacity {4196.8 [J/(kg K)]}, and d*T*/d*t* is the initial slope of the temperature curve. A representative example of the fitting curve used to calculate local SAR is shown in Figure 2B. For temperature measurements, a fiber-optic thermometer (Luxtron One, Lumasense Technologies, CA, United States, accuracy ±5%), with a tip having an approximated volume of 0.5 mm^3^ was used. The probe was vertically deepened, at the center of the MEA, in the culture medium to ensure contact with the bottom of the culture chamber where neurons are located during the exposure. Reproducibility was assured by at least three measurements per condition.

Both measurements and simulations were performed without cells since a thin cell monolayer does not significantly affect local SAR distribution.

### 2.6. Protocol for cells RF exposure

Neuronal cultures were exposed between 17 and 27 days *in vitro* (DIV). In this range, the neuronal activity is balanced between random spikes and bursts (18, 19). In all experiments, after placing the MEA in the recording setup, we waited for the stabilization of the temperature inside the incubator to work under standard cell culture conditions (37°C, 5% CO_2_). The experimental protocol lasted 75 min and was divided into 5 phases of 15 min, including two pre-exposure (S1 and S2), one exposure (E), and two post-exposure phases (P1 and P2), as schematically represented in Figure 1D. Sham exposures were carried out using the same protocol but with no RF-EMF emitted. During the RF exposure phase (E), we used both CW and 5G-modulated 3.5 GHz RF signals at SAR values of 1 or 3 W/kg, slightly below or above the ICNIRP guidelines for local exposure.

We also assessed the effects of high-level signals (SAR of 28 W/kg) as we previously observed an inhibitory effect of CW 1.8 GHz-RF exposure at 28 W/kg (10). In this previous study, the experimental conditions differed on one point: the culture medium was continuously perfused. Therefore, to characterize the role of carrier frequency and compare experiments under identical conditions, we also conducted RF exposure with a 1.8 GHz CW signal at 28 W/kg, with the setup described in the section “RF exposure system.”

Each culture was exposed to only one SAR exposure condition (1 or 3 or 28 W/kg). To guarantee the reproducibility of results, each experiment was carried out at least 8 times. The exact number of experiments for each exposure condition is detailed in the figure caption.

### 2.7. Exposure of cells to heat

To assess the role of temperature elevation during RF exposure, neuronal cultures were also subjected to direct heating in the absence of RF. To perform these experiments, the same five-phase protocol (as described above) was used but the E phase corresponded to heating the culture medium using a warming plate located underneath the MEA. The heating was regulated by a specific controller (TC01, MCS) that was adjusted to reach the temperature increase caused by RF-induced heating in the culture medium at 28 W/kg.

### 2.8. Choice of metrics and data analysis

To characterize the neuronal activity and quantify RF-induced changes in the network activity, four metrics were assessed for each recording phase. Since neuronal cultures exhibit activity patterns that include both spikes and bursts, we used metrics characterizing either spiking or bursting activity.

1) Mean firing rate (MFR)

(2) $$\mathrm{MFR}=\frac{\sum \mathrm{FR}}{N_{S\;\left(S1\right)}}$$

where FR is the firing rate, i.e., the total number of spikes per second collected over all active electrodes, and *N*_S_ (S1) is the total number of active electrodes in terms of spiking activity in the S1 phase. *N*_S_ was calculated by considering an electrode active when its spike rate was at least 0.1 Hz.

2) Mean bursting rate (MBR)

(3) $$\mathrm{MBR}=\frac{\sum \mathrm{BR}}{N_{B\;\left(S1\right)}}$$

where BR is the bursting rate, i.e., the total number of bursts per minute collected over all active electrodes, and *N*_B_ (S1) is the total number of active electrodes in terms of bursting activity in the S1 phase. *N*_B_ was calculated by considering an electrode active when its burst rate was at least 0.04 Hz.

3) Mean burst duration (MBD)

(4) $$\mathrm{MBD}=\frac{\sum \mathrm{BD}}{N_{B}}$$

where BD is the burst duration defined as the average burst duration calculated across all active electrodes.

Mean outside bursts firing rate ( $M_{\mathrm{OB}}\mathrm{FR})$

(5) $$M_{\mathrm{OB}}\mathrm{FR}=\frac{\sum_{\mathrm{OB}}\mathrm{FR}}{N_{B\;\left(S1\right)}}$$

where _OB_FR is the outside bursts firing rate, i.e., the total number of isolated spikes (occurring outside bursts) per second collected over all active electrodes.

To properly characterize the effect of RF exposure on the entire neuronal network, we only considered the number of active electrodes in the S1 phase. Moreover, to deal with the basal electrical activity variability, these four metrics were normalized by considering the S1 phase as 100%. Multiple comparison tests were performed using Kruskal–Wallis, followed by Conover’s post-hoc test. Statistical analyses were performed with R (20) and the PMCMRplus (21) package.

## 3. Results

### 3.1. Numerical and experimental dosimetry at 3.5 GHz

Figure 2A shows the SAR distribution at 3.5 GHz normalized to 1 W incident power along a vertical cut across the TEM cell and the MEA. The SAR is locally homogeneous at the level of the neurons, which are at the bottom of the MEA. The local SAR value is averaged over 0.5 mm^3^ of the exposed culture medium, corresponding to the volume of the thermal probe, as depicted in Figure 2A. Simulated local and whole-volume SAR values for 1 W input power were 31.2 W/kg and 77.5 W/kg, respectively.

Figure 2B shows the measured temperature elevation of the culture medium when exposed to 1 W incident power. Experimental local SAR normalized to 1 W incident power was equal to 28 ± 4 W/kg, a value consistent with numerical simulations. Based on this value, input TEM cell power during exposure was adjusted to 15.5 dBm, 20.3 dBm, 30 dBm, for SAR levels of 1, 3, or 28 W/kg, respectively.

### 3.2. Thermal dosimetry at 3.5 GHz and direct heating

**Figure 3:**
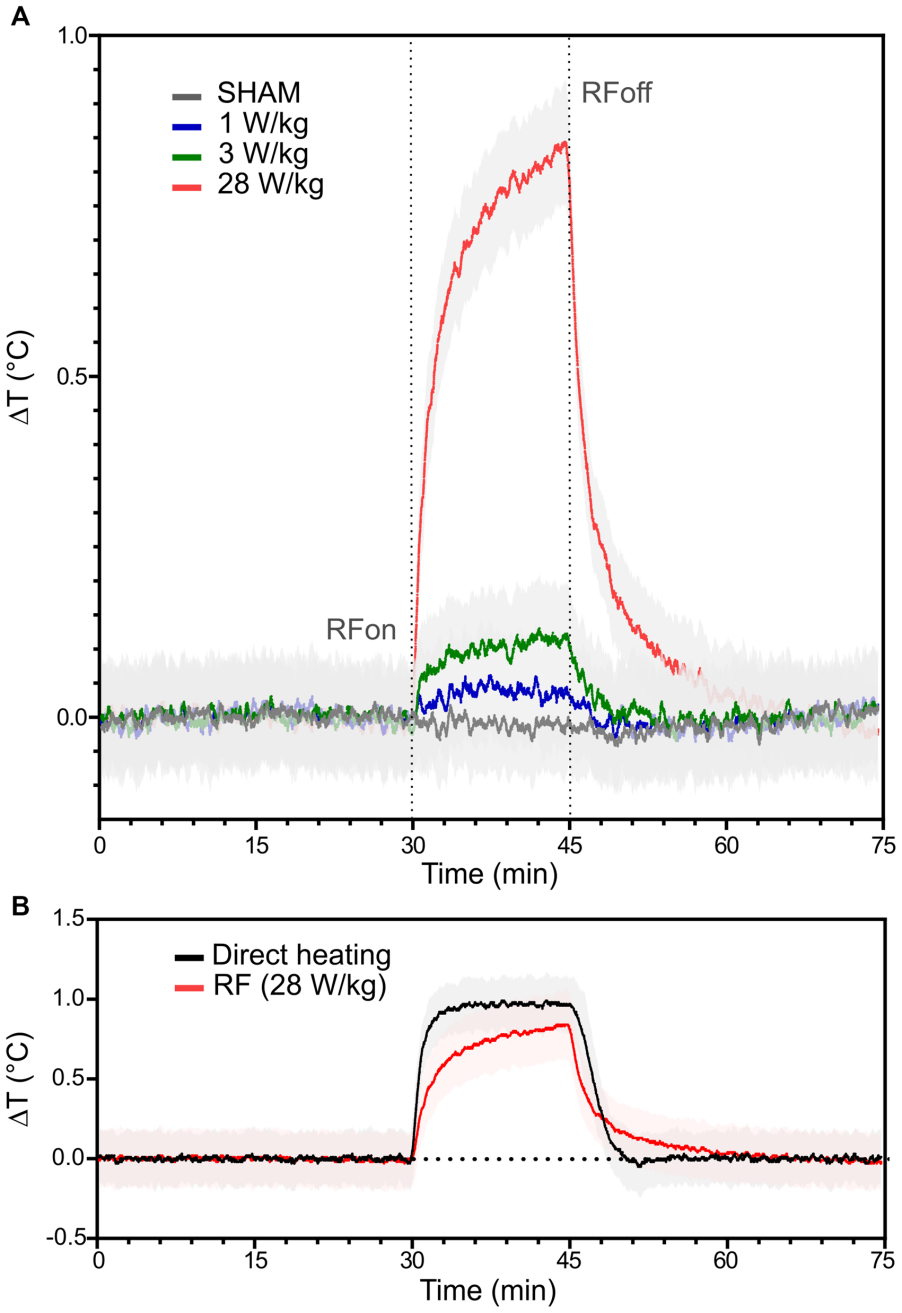
RF- and warming plate-induced temperature elevation in the neuronal culture medium. **(A)** RF-induced temperature increase of the culture medium when exposed to different SAR levels; 0 (*n* = 5), 1 (*n* = 5), 3 (*n* = 5), and 28 W/kg (*n* = 6) are, respectively, plotted in grey, blue, green and red. Black dotted lines indicate the beginning (RF_on_) and the end (RF_off_) of the RF exposure phase. **(B)** Representation of temperature rises induced by RF exposure at 28 W/kg (in red; *n* = 6) or direct heating using the warming plate (in black; *n* = 3). The solid traces represent the average temperature elevation, while the light areas represent the standard deviation.

The temperature of the culture medium was measured along the 5 phases of the experimental protocol (Figure 1D). Figure 3A shows the RF-induced temperature elevation during the E phase when the culture medium was exposed to CW 3.5 GHz signal for 15 min. The initial slope of the RF-induced temperature rise (at *t* = 30 min) and the temperature achieved at the end of the exposure phase (at *t* = 45 min) are dependent on the SAR level. The average temperature increase was 0.06°C, 0.13°C, and 0.80°C at 1 W/kg, 3 W/kg, and 28 W/kg, respectively (Figure 3A). As expected, maximal temperature elevation was achieved under RF exposure with a SAR of 28 W/kg. In Figure 3B, we compare the temperature rise induced in the culture medium by CW exposure at 28 W/kg or by direct heating using the warming plate. We have performed 6 measurements of temperature elevation during RF exposure at 28 W/kg. At the end of the 15 min RF exposure, the average temperature rise was (0.8 ± 0.2) °C. For experiments with direct heating, we conducted 3 temperature measurements. At the end of the 15 min heating time, the average temperature rise was (1.0 ± 0.1) °C. We obtained curves with small differences in terms of temperature dynamic and amplitude, notably in rising and falling transient phases.

### 3.3. RF exposure of cells at 3.5 GHz

Neuronal cultures were exposed to a 3.5 GHz-RF signal at increasing SAR levels of 1, 3, and 28 W/kg. For all exposure conditions, the electrophysiological recordings of the RF-exposed phase (E) were compared with the pre-exposure (S1, S2) and the post-exposure (P1, P2) recordings, according to the four metrics defined in “Choice of metrics and data analysis” section: MBR, MFR, MBD, and M_OB_FR.

#### 3.3.1. Exposure at SAR levels of 1 and 3 W/kg

**Figure 4:**
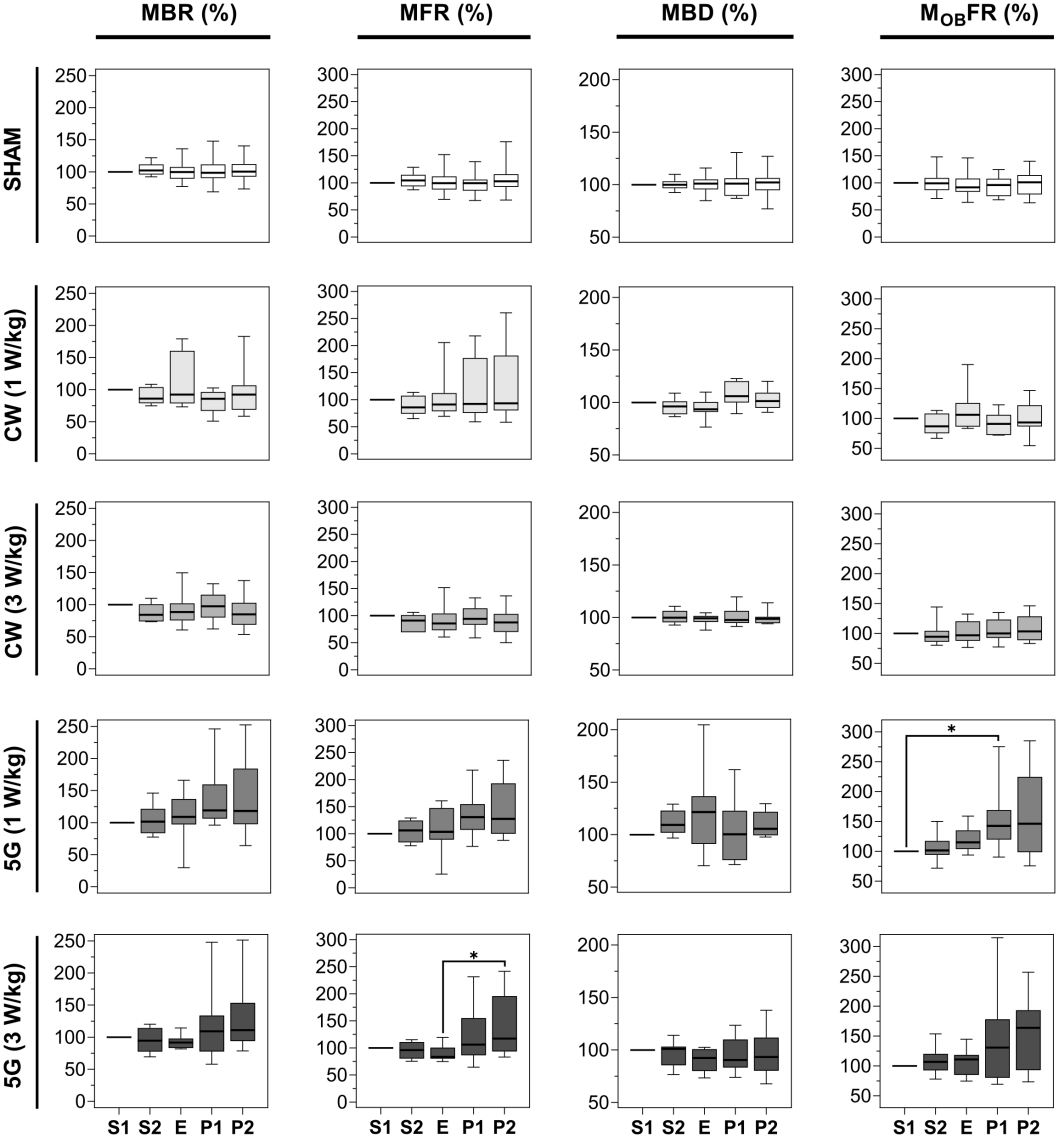
Statistical analysis of the effects of 15 min RF exposure at 3.5 GHz CW and 5G-modulated signals at SAR of 1 and 3 W/kg on the electrical activity of cultured cortical networks. Box plots are representative of the mean bursting rate (MBR), the mean firing rate (MFR), the mean burst duration (MBD), and the mean firing outside bursts rate (M_OB_FR) measured along the 5 phases (S1, S2, E, P1, P2) of the sham or RF-exposure protocols. Data are normalized considering the S1 phase as 100%. From top to bottom: sham-exposed cultures (*n* = 19); RF-exposed cultures to CW signals at a SAR of 1 W/kg (*n* = 8); RF-exposed cultures to CW signals at a SAR of 3 W/kg (*n* = 9); RF-exposed cultures to 5G signals at a SAR of 1 W/kg (*n* = 8); RF-exposed cultures to 5G signals at a SAR of 3 W/kg (*n* = 9). (^*^p ≤ 0.05, Conover multiple comparison test).

We first analyzed the effect of 15 min RF exposure to 3.5 GHz CW and 5G-modulated signals at SAR of 1 and 3 W/kg. Figure 4 shows the box plots illustrating the four metrics evaluated in each exposure condition, including the sham exposure (SHAM). For the sham experiments, the chosen parameters are extremely stable between the different phases. For CW experiments, there were no significant differences between the non-exposed phases (S1, S2, P1, and P2) and the RF-exposed phase (E) for all metrics considered and whatever SAR used (*p* > 0.05). For 5G modulated experiments at 1 and 3 W/kg, the MBR and the MBD were not different between the 5 phases. However, when neuronal networks were exposed to 5G-modulated signals at 1 W/kg, the M_OB_FR increased significantly during the first post-exposure phase (P1) compared to the first pre-exposure phase (S1). The MFR also increased slightly between the E and P2 phases in cell cultures exposed at 3 W/kg (*p* < 0.05).

#### 3.3.2. Exposure at a SAR level of 28 W/kg

**Figure 5:**
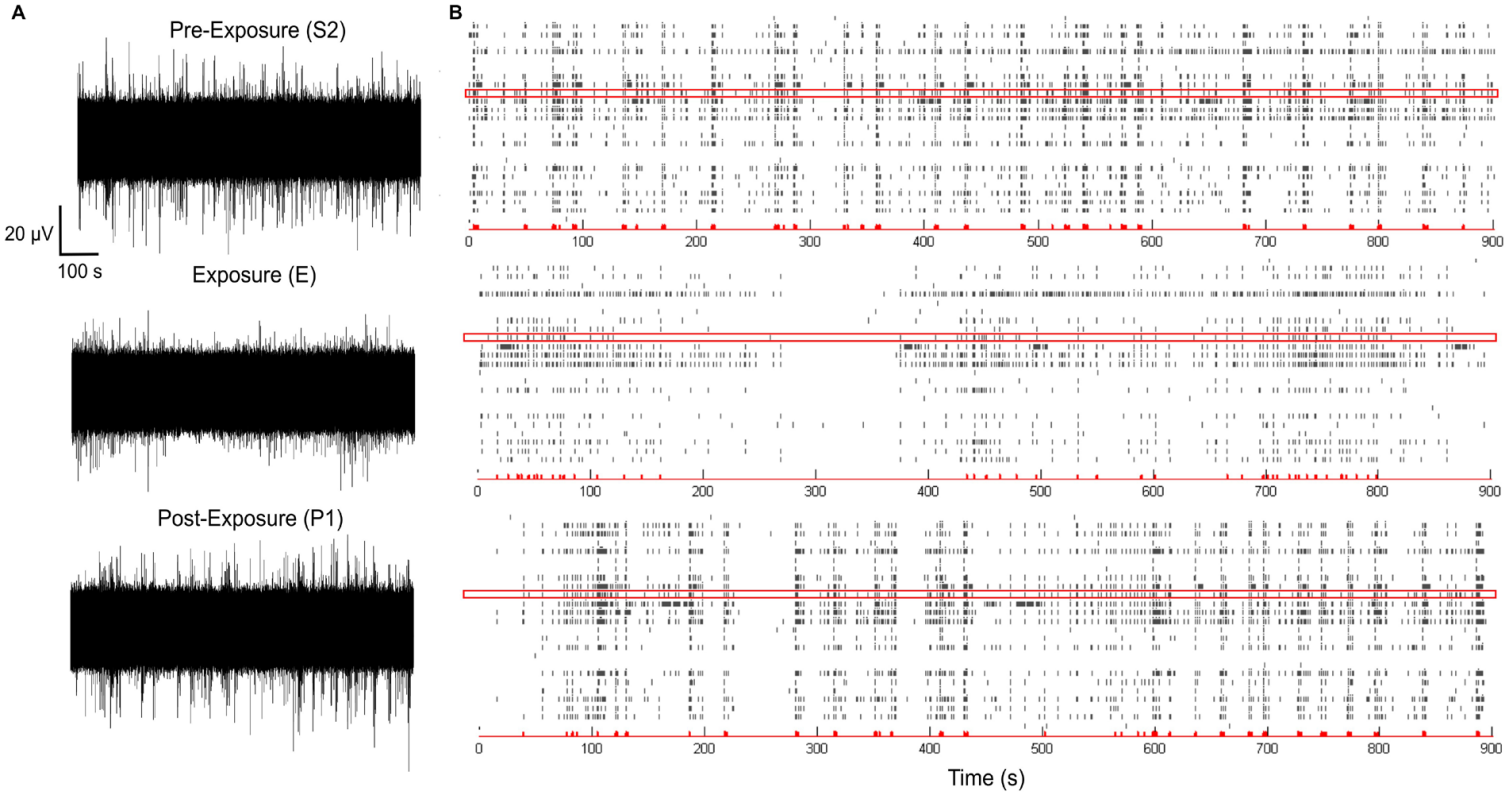
Representative example of spontaneous electrophysiological activities of neuronal networks recorded by a multi-electrode array (MEA). **(A)** Spontaneous electrical activity of a neuronal culture recorded over 15 min on a single electrode before (S2 phase), during (E phase), and after (P1 phase) exposure to 5G-modulated 3.5 GHz signals at 28 W/kg. **(B)** Corresponding raster plots for each phase showing the analyzed electrical activity of neurons on 24 electrodes (each one is represented by a horizontal line). The red box corresponds to the electrode shown in **(A)**.

We next assessed how exposure to 3.5 GHz signals (CW and 5G) at a SAR value of 28 W/kg impacted the electrical activity of the neurons. While this SAR value is well above the guidelines, it corresponds to the level used in our previous exposure experiment at 1.8 GHz (10). Figure 5 shows representative examples of the recorded S2, E, and P1 phases and corresponding raster plots during exposure to 5G signals at 28 W/kg. These recordings revealed an apparent decrease in the electrical activity of neurons during the exposure phase (E) that progressively disappeared in the first minutes of the P1 phase.

**Figure 6:**
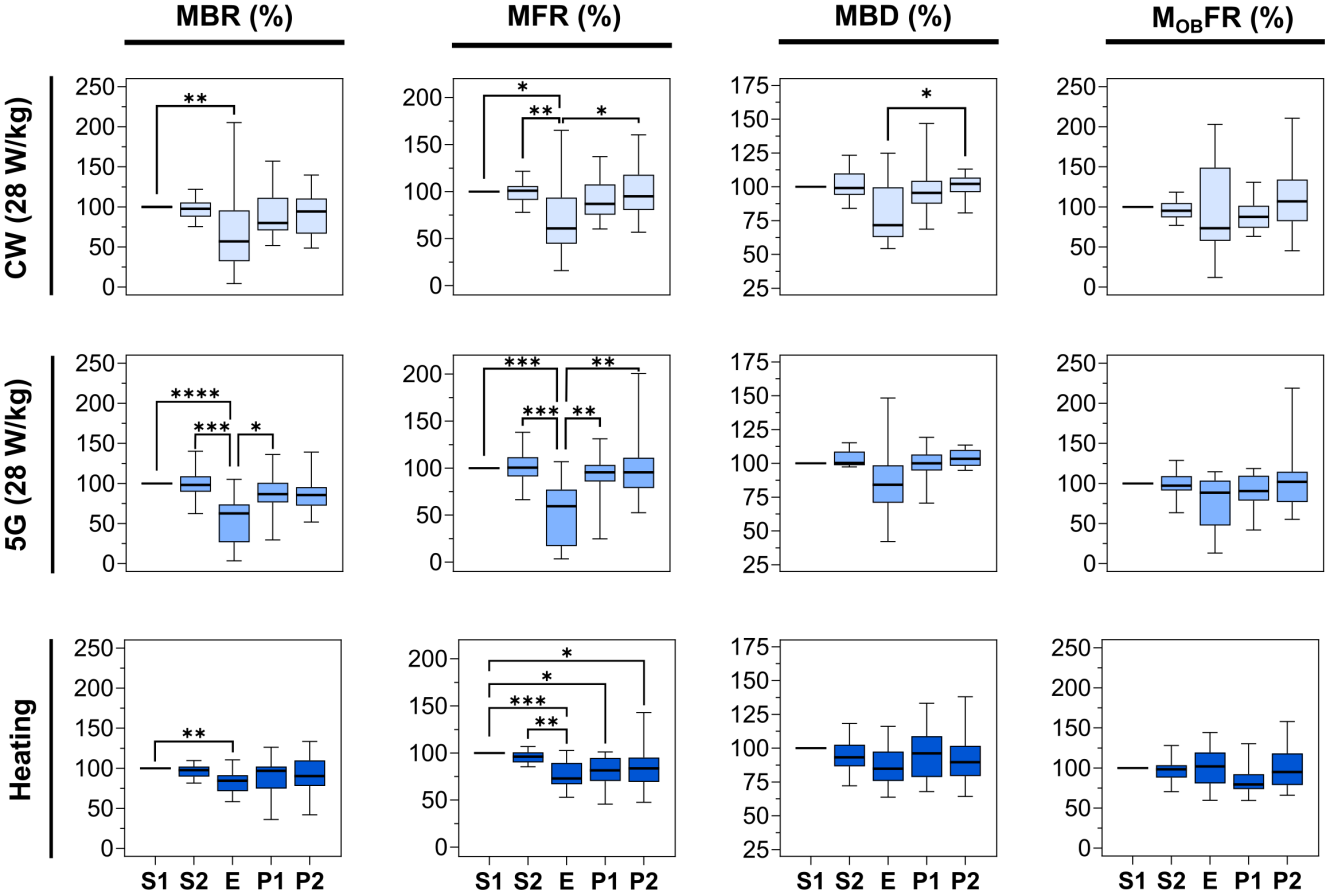
Statistical analysis of the effects of 15 min RF exposure at 3.5 GHz CW and 5G-modulated signals at a SAR of 28 W/kg and warming plate equivalent heating on the electrical activity of cultured neuronal networks. Box plots are representative of the mean bursting rate (MBR), the mean firing rate (MFR), the mean burst duration (MBD), and the mean firing outside bursts rate (M_OB_FR) measured for the neuronal cultures exposed either to RF or direct heating, along the 5 phases (S1, S2, E, P1, P2). Data are normalized considering the S1 phase as 100%. From top to bottom: RF-exposed cultures to 3.5 GHz CW signal at a SAR of 28 W/kg (*n* = 16); RF-exposed cultures to 5G-modulated 3.5 GHz signal at a SAR of 28 W/kg (*n* = 14); cell cultures heated by thermal conduction (1°C rise) (*n* = 13) using a warming plate (^*^p ≤ 0.05, ^**^p ≤ 0.01, ^***^p ≤ 0.001, ^****^p ≤ 0.0001, Conover multiple comparison test).

The first two lines of Figure 6 illustrate the normalized parameters (MBR, MFR, MBD, and M_OB_FR) for 16 CW-and 14 5G-exposed cultures at a SAR of 28 W/kg. In all experimental conditions, the four parameters were stable along the two pre-exposure phases (S1 and S2). When neuronal cultures were exposed to CW or 5G-modulated signals at 28 W/kg, both the MBR and the MFR were significantly reduced in the E phase in comparison to the S1 phase. These two parameters also appeared to be significantly different between the S2 and E phases except for the MBR in the CW-exposure condition for which we only observed a decreasing trend. Whatever the conditions, there were no statistically significant differences between the pre-exposure phases (S1, S2) and the post-exposure phases (P1, P2), indicating that the inhibition in bursting and firing rate during exposure was reversible. However, depending on the conditions and metrics considered, the exposure phase (E) was not systematically different from the post-exposure phases (P1, P2). For instance, in the CW-exposure condition, the MFR of the E-phase is not different from the MFR of the P1-phase.

To quantify the magnitude of the effect, the median values were compared between phases. For the CW exposure, the MBR decreased by 43.03% during the E-phase compared to the S1 phase (*p* < 0.01). The MFR also decreased by 39.11% and 40.01% in comparison to S1 (*p* < 0.05) and S2 phases (*p* < 0.01), respectively. For 5G-modulated signals, the MBR during the exposure was reduced by 37.42% and 35.54%, respectively, in comparison to S1 (*p* < 0.0001) and S2 (*p* < 0.001) phases. The MFR also decreased by 40.59% and 41.19% compared with S1 (*p* < 0.001) and S2 (*p* < 0.001) phases. In sharp contrast, the MBD and the M_OB_FR did not change significantly between phases, except for the MBD which was significantly different between E and P2-phases (*p* < 0.05) in CW experiments. Globally, even if these two parameters are not significantly different between phases, they tend to decrease in the E phase.

**Figure 7:**
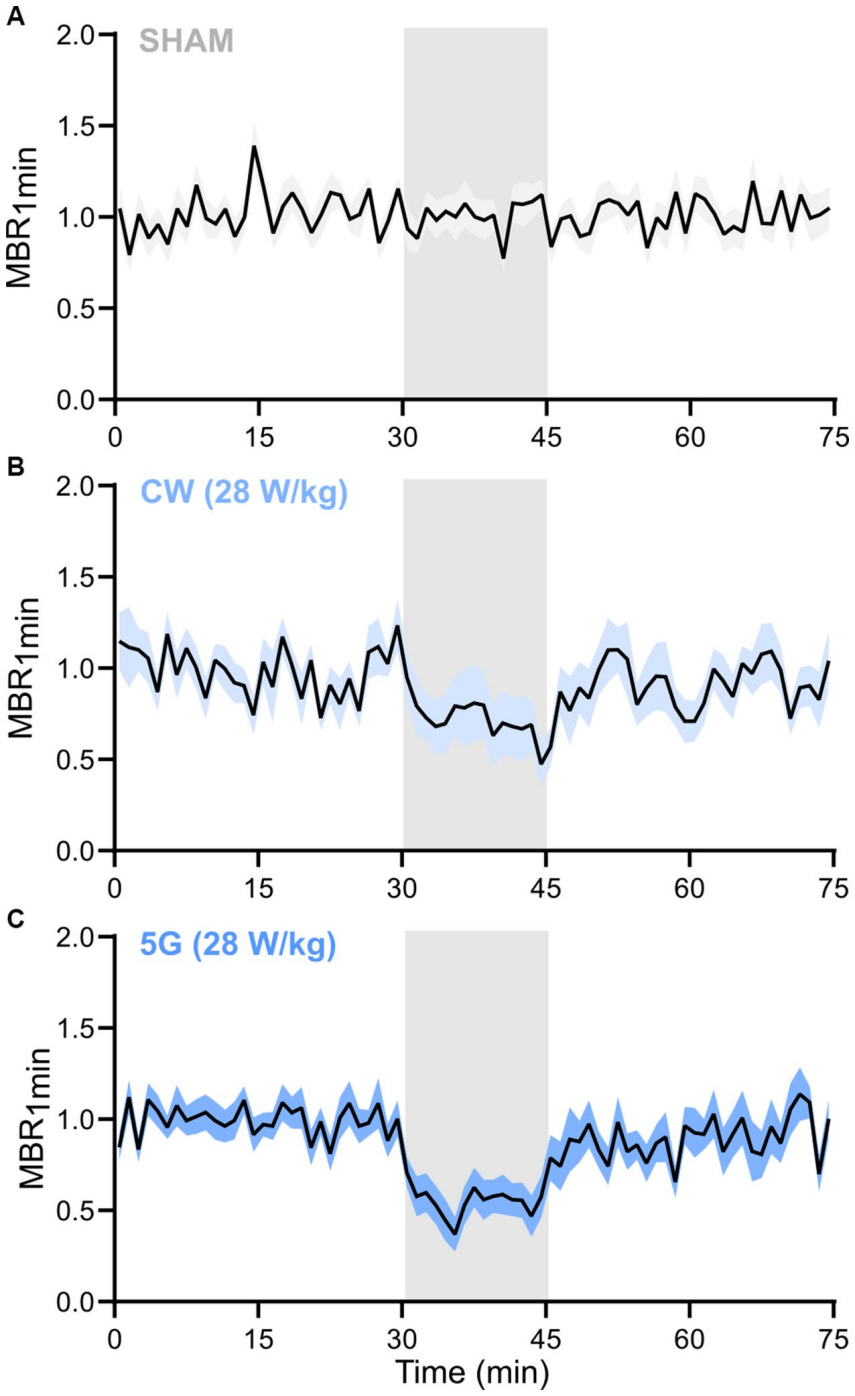
Temporal dynamic of the mean bursting rate along the RF exposure protocol to 3.5 GHz signals at 28 W/kg. Normalized temporal time course of the mean bursting rate averaged over 1 min (MBR_1 min_) along the 75 min of the experimental protocol for **(A)** SHAM (*n* = 19), **(B)** CW signals (*n* = 16), and **(C)** 5G signals (*n* = 13) groups of runs (data are shown as mean ± SEM). The RF exposure phase is represented by a grey-shadowed area (RF_on_ at 30 min and RF_off_ at 45 min).

Specific inhibition of the neuronal activity was further analyzed by measuring the temporal variation of the MBR_1 min_, i.e., the MBR averaged over 1 min of recording along the whole experiment lasting 75 min and averaged over the total number of experiments, for each condition. While the MBR_1 min_ tends to be relatively stable over time in the sham experiment (Figure 7A), an important decrease in the MBR_1 min_ was observed at the beginning of the exposure to the 5G-modulated 3.5 GHz signal at 28 W/kg (Figure 7C). Indeed, the MBR_1 min_ dramatically decreased by over half of the baseline level during the first minutes of exposure and then stabilized. For CW exposure, the MBR_1 min_ also decreased over time until reaching almost 50% inhibition at the end of the 15 min exposure phase (Figure 7B). As expected regarding the results of averaged metrics (Figure 6), the MBR_1 min_ gradually returned to baseline after the RF signal was turned off.

### 3.4. Thermal exposure of cells without RF

We finally assessed whether the moderate temperature increase (<1°C) measured during RF exposure to 3.5 GHz signals at 28 W/kg could be responsible for the decrease in neuronal electrical activity. Thus, we exposed neuronal cultures to direct heating during the E phase, with the temperature profile shown in Figure 3B. Results on neuronal activity are illustrated in Figure 6. Interestingly, we observed an effect, especially on the MBR and the MFR. While S1 and S2 phases were stable, the MBR and the MFR were significantly reduced in the heating phase (E) by 15.66 and 27.11% compared to the S1 phase, respectively. Moreover, the MFR for the P1 and P2 phases were significantly lower than in the S1 phase (*p* < 0.05), indicating that the spike inhibition was not reversible.

### 3.5. RF exposure of cells at 1.8 GHz and a SAR level of 28 W/kg

Whether RF exposure to 3.5 GHz signals induces comparable effects on the electrical activity of neurons to those observed at 1.8 GHz was then tested using our exposure system (see “RF exposure system” section and Figure 1).

**Figure 8:**
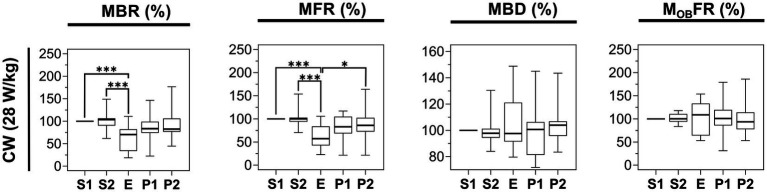
Statistical analysis of the effects of 15 min RF exposure with CW signal at 1.8 GHz at 28 W/kg on the electrical activity of cultured cortical networks. Box plots are representative of the mean bursting rate (MBR), the mean firing rate (MFR), the mean burst duration (MBD), and the mean firing outside bursts rate (M_OB_FR) measured along the 5 phases (S1, S2, E, P1, P2) of the experimental protocol. Data are normalized considering the S1 phase as 100%. Cells were exposed to 1.8 GHz CW signals at a specific absorption rate (SAR) of 28 W/kg (*n* = 15) (^*^p ≤ 0.05, ^**^p ≤ 0.01, ^***^p ≤ 0.001, Conover multiple comparison test).

Since we observed an effect of 3.5 GHz exposure at 28 W/kg, we assessed whether the carrier frequency was important in generating this inhibitory effect. Using the same setup, we exposed neuronal cultures to a 1.8 GHz CW RF signal at a SAR of 28 W/kg. As shown in Figure 8, comparing E and S1 phases, we obtained a significant decrease of 29.6% of the MBR and 42.93% of the MFR during exposure. We also studied the effect of such exposure on other parameters characterizing the electrical activity of neurons, such as the MBD and the M_OB_FR, but we did not detect any exposure-related changes in these parameters.

## 4. Discussion

In the present study we assessed the effects of RF exposure at 3.5 GHz on the electrical activity of rat cortical neuronal networks *in vitro* at different SAR levels. Our results showed that exposure to 3.5 GHz CW and 5G-modulated signals for 15 min at SAR levels ranging from 1 to 3 W/kg do not cause major changes in firing and bursting activities. In sharp contrast, at high SAR level of 28 W/kg, 15 min exposures to both 3.5 GHz CW and 5G-modulated signals clearly inhibited the spontaneous activity of the neuronal networks. At this specific high SAR level, both mean spiking (MFR) and bursting (MBR) activities were decreased by ~40%, while the burst duration (MBD) and the number of spikes outside bursts (M_OB_FR) remained stable throughout the entire experiment, suggesting an effect on burst generation. We should note that this inhibitory effect was reversible. Additionally, the RF signal modulation had no impact on the magnitude of the effect, as inhibition was comparable in experiments with CW or 5G signals (Figure 6). When replicating the experiment with CW 1.8 GHz exposure, similar decreases of ~40% of the MFR and ~30% of the MBR were observed, while both the MBD and the M_OB_FR remained unchanged (Figure 8). These findings suggest that the carrier frequency does not influence the amplitude of the effect in the 1.8–3.5 GHz range. Furthermore, the present findings at 1.8 GHz are in agreement with our previous published work (10), which showed a comparable decrease of ~35% of the MBR under CW exposure at the same SAR level, while the culture medium was then continuously and slowly perfused. This indicates that the circulation of thermostated culture medium during the 75 min experiment is not necessary to induce this effect.

In summary, we found an inhibitory effect of RF exposure on neuronal networks *in vitro*. However, the threshold for this inhibitory effect exceeds the SAR limit recommended by ICNIRP for environmental exposure (1). Interestingly, our study also indicates a specific effect on bursts while the number of isolated spikes (outside bursts) remained stable. This prompted us to further investigate the underlying mechanisms responsible for burst generation in neuronal cultures.

### 4.1. Electrical activity of dissociated neuronal cultures

Cultures of dissociated neurons generally follow a relatively reproducible developmental pattern. After a few days of culture, spontaneous electrical activity appears as isolated and unsynchronized spikes. Starting on the second week of culture, there is a gradual evolution towards bursts of activity occurring synchronously across multiple active sites. The synchronism of these bursts is progressively reinforced over time, while at the same time, the spiking activity tends to be completely abolished. This sequential pattern of electrical signals seems to be a common feature of dissociated cultures of neurons and is also found in different neuronal tissues (22–25). It has been shown that changes in this spontaneous electrical activity are correlated with topology and morphology changes in the network occurring during its development (26, 27). Moreover, these changes are also closely associated with homeostasis and long-term synaptic plasticity (28) and their origin results from different causes (29).

At the cellular level, it was shown that some cortical cells exhibit specific intrinsic properties, making them spontaneously generate rhythmic activity (30). These pacemaker properties are well known and related to the interaction of T-type calcium and Ih currents and may play a role in the network’s spontaneous activity. Finally, the spontaneous firing of neuronal assemblies can also cause long excitatory post-synaptic potential (EPSP) mediated by NMDA receptors (29, 31) and modulated by AMPA receptors (32) initiating bursts.

At the network level, a local confluence of action potentials generation can trigger activity in the target neurons producing bursts (33). This transmission is mediated by chemical synapses, which are the major actors in the propagation of electrical activity. The strength of chemical synapses is modulated by pre-and post-synaptic activity. It is also under the control of a spontaneous form of neurotransmission-termed miniature currents (34) and increased excitatory synaptic strength generally facilitates the propagation of bursts through the network.

As the bursting activity pattern results from several mechanisms related to the dynamics of excitatory and inhibitory synapses (26), its decrease under RF exposure suggests that RF alters the overall excitatory/inhibitory balance in the network.

### 4.2. Effects of EMF exposure on neuronal networks *in vitro*

Many *in-vitro* studies have also assessed whether RF exposure impact neuronal activity. For instance, an experiment combining an open TEM cell with 60-electrode MEAs (35) was carried out to assess the effects of pulsed TETRA RF signals on cortical neuronal networks (36). The carrier frequency was 395 MHz pulsed at 17.64 Hz (TETRA signal). The authors reported no significant difference in the bursting rate before and after a series of 15 min exposures at 1.17 and 2.21 W/kg. However, the experimental device was not set up to detect electrical activity during the exposure phase, which prevented them from detecting reversible alterations. Xu et al. (37) investigated the impact of chronic RF exposure on synaptic function of cultured hippocampal neurons of rats using the patch-clamp technique in association with immunohistochemistry. In that study, cell cultures were subjected to 15 min of daily exposure (DIV7–DIV14) to GSM 1.8 GHz RF signals at 2.4 W/kg. RF exposure selectively reduced the AMPA miniature excitatory post-synaptic currents (mEPSCs) amplitude, whereas the frequency of AMPA mEPSCs as well as the NMDA mEPSCs current amplitude remained unaffected. This group also noted that the number of (PSD95)-stained puncta significantly decreased in RF exposed neurons, suggesting a reduction in the number of excitatory synapses. More recently, another *in-vitro* investigation conducted by Echchgadda et al. (31) reported the impact of CW 3.0 GHz RF exposure on neuronal excitability in cultured primary hippocampal neurons. Whole-cell patch-clamp recordings were performed within 30 min after exposing the neurons at low SAR levels of ~0.3 W/kg, for 60 min. Results revealed a significant depolarization of the neuronal resting membrane potential after exposure, making neurons more excitable. This effect was also associated with decreased action potential (AP) amplitude. In addition, the exposure enhanced synaptic transmission leading to an increase in the amplitude of glutamate-dependent spontaneous excitatory post-synaptic currents (sEPSCs) and GABA-dependent spontaneous inhibitory post-synaptic currents (iPSCs).

The variability of outcomes among our study and those obtained by Xu et al. (37) and Echchgadda et al. (31) may be attributed to differences in the experimental approach such as neuronal subtypes, duration of exposure (short-term versus chronic), carrier frequency, SAR values and the possibility to assess the neuronal activity during RF exposure. However, all these studies point to a possible non-thermal effect of RF on the balance between excitatory and inhibitory synapses.

Additionally, several research reports described the impact of millimeter waves (MMW, 30–300 GHz) on neuronal cells. These studies present renewed interests in the context of the deployment of 5G technology, which is based in part of the use of the 26 GHz frequency band. One investigation conducted by Pikov et al. (38) assessed the impact of MMW 1 min exposures (60.125 GHz) on neuronal firing using cortical slices and patch-clamp experiments. In cells exposed for 1 min at power densities of 1 μW/cm^2^, the firing rate of individual pyramidal neurons decreased in 4 of the 8 tested neurons. This effect was associated with the narrowing of the AP and the decrease in membrane resistance. The same research group also examined the effects of low-power 60 GHz MMW on individual neurons in the leech ganglia, a simpler biological model. In agreement with their previous study, they found that exposure to MMW tends to decrease the firing rate of neurons (39). Even if these studies were not performed in the same frequency range, the results are in line with those obtained in the present work.

### 4.3. Influence of temperature

Knowing that 3.5 GHz RF exposures at 28 W/kg induced a local temperature elevation of ~1°C, we decided to characterize the effect of direct heating on neuronal electrical activity. Interestingly, we found that thermal exposure also reduced the MFR and the MBR by approximately 27% and 15%, respectively, which is about half the decrease observed using RF exposure. Furthermore, in contrast to RF exposure at 28 W/kg, for the MFR alone, inhibition by direct heating was not immediately reversible in the 30 min post-exposure period (Figure 6). These results suggest that the RF-induced decrease of neuronal electrical activity observed at 28 W/kg cannot be entirely attributed to the concomitant temperature elevation.

Other research groups have also compared the effects induced by RF-EMF exposure and equivalent direct heating on neuronal activity. For instance, Romanenko et al. (39) performed experiments on individual neurons in the leech ganglia using microelectrode techniques. They found that low-level (1, 2, and 4 mW/cm^2^) 60 GHz MMW exposure and gradual bath heating (from around 21–22°C up to 27°C) of ganglionic neurons produced nearly identical effects on the resting membrane potential and the AP amplitude. However, the firing rate was slightly suppressed during MMW exposure at all applied power densities but increased in a dose-dependent manner during gradual bath heating. We can note that the heating rate was 0.59°C/min during the MMW irradiation and 2.4°C/min during bath heating. More recently, the same group (40) investigated the effect of higher power MMW (60 GHz, >100 mW/cm^2^) on primary sensory neurons from the medicinal leech. Such neurons are only activated when exposed to painful stimulus (pressure, chemical or osmotic agent, temperature changes). Authors demonstrated that electrophysiological responses of these sensory neurons differed between MMW exposure and direct heating. Indeed, bath heating of sensory neurons evoked spike trains with higher spiking rate when compared to MMW irradiation. However, the authors noted that the average temperature rise rate was different: 6.6°C/min for MMW and 12.6°C/min for direct conductive heating. In our study, we also had differences in the rates of temperature rise between RF exposure and direct heating (Figure 3). Indeed, the measured temperature rise rates for RF exposure and direct heating were equal to 0.09 and 0.38°C/min, respectively. In further studies, it will be interesting to perform experiments using different rates of temperature rise, while assessing corresponding changes in neuronal activity.

Independently of RF exposure, numerous studies have investigated the impact of temperature elevation on neuronal activity, both in terms of temperature increment and rate of change. For instance, Kölher et al. (36) found that raising the temperature from 37 to 37.5°C at 0.1°C/min increased spike and burst rates of cortical neurons *in vitro*. However, the results were not statistically significant. Our group has also demonstrated that increasing by 1°C the temperature of the culture medium led to an increase in the bursting activity of cortical neurons. These experiments were conducted by gradually elevating the temperature by approximately 0.16°C/min (8). More recently, Odawara et al. (41) investigated the effect of increasing the temperature from 37°C to 46°C on the spontaneous activity of hiPSC-derived sensory neurons. They used a heating rate of 1.4°C/min and observed that the firing frequency increased with the temperature, particularly above 40°C. In contrast, Newberry et al. (42) did not observe any temperature-dependent effect on the spiking rate when recording spontaneous activity in dorsal root ganglia (DRG) rat neurons at 37°C and 42°C. Taken together, the findings of these studies suggest that an elevation in temperature generally led to an increase in the firing rate of neurons. However, these experiments were carried out under various experimental conditions with different temperature rise rates. Neurons might not be sensitive to the temperature elevation itself but rather to the time profile of the gradual temperature increase as supported by several studies. For instance, Bolshakov and Alekseev (43) showed that changes in the electrical activity of pacemaker neurons of the pond snail were dependent on the heating rate. Indeed, they noticed that a rapid temperature increase caused inhibition of the firing rate (due to fast activation of the sodium pump), whereas slow warming had the opposite effect by increasing the firing rate. Moreover, Alekseev et al. (44) found that MMW exposure produced biphasic changes in the firing frequency of neurons. Interestingly, this biphasic alteration was reproduced through the application of heat, provided that the magnitude and rate of temperature rise were comparable to those observed during MMW exposure. Overall, these findings highlight that we cannot exclude that the rate of temperature change could be of importance in defining the neuronal response to RF or thermal exposure.

### 4.4. Influence of the electrodes

Special care has been paid to the electromagnetic compatibility of our experimental bench which combines *in vitro* electrophysiology and RF exposure. The influence of electrodes at the bottom of the culture well and their possible interference with EMF has already been discussed in our previous studies (7, 8). Despite shielding, the maximal voltage that could remain on the electrodes (1 mV) was not likely to alter the electrophysiological activity of the neuronal network. Furthermore, in our more recent experiments (10) as well as in the present work, we used a new type of MEA that was custom designed to further improve the electromagnetic compatibility, mainly resizing the aperture for optical visualization and adding ground planes in the multi-layer PCB (9). However, it would still be useful to explore alternative methods to electrophysiology to characterize the spiking activity of neuronal networks *in vitro* under RF exposure.

### 4.5. Conclusion

In conclusion, we have given here experimental evidence that RF exposure of cultured cortical neurons at 3.5 GHz CW or 5G-modulated signals at 28 W/kg induces a decrease in total firing and bursting activities. The threshold for such inhibitory effect exceeds the maximal SAR recommended by ICNIRP for human exposure (1). Considering the studies mentioned above, we may hypothesize that the rate of the temperature rise plays an important role in eliciting specific cellular responses. Further studies are needed to elucidate the role of temperature rate and thus investigate the mechanism underlying these observations.

## Data availability statement

The original contributions presented in the study are included in the article/supplementary material, further inquiries can be directed to the corresponding author.

## Ethics statement

The animal study was reviewed and approved by the Bordeaux Ethics Committee for Animal Experimentation (CEEA-050).

## Author contributions

NL, YP, PL, DA-C, and IL participated in research design. AC, FPdG, RO, and PL conducted experiments. AC, AG, NL, RO, PL, and YP performed data analysis. AC, NL, YP, RO, AG, FPdG, IL, PL, and DA-C wrote or contributed to the writing of the manuscript. All authors contributed to the article and approved the submitted version.

## Funding

This research was funded by the French Agency for Food, Environmental and Occupational Health and Safety (ANSES) under grant agreement EST-2019 RF-18 (The 5G-SAMU Project) and the New Aquitaine regional council under grant agreement AAPR2020A- 2019-8140010 (The PHYSTRIG project).

## Conflict of interest

The authors declare that the research was conducted in the absence of any commercial or financial relationships that could be construed as a potential conflict of interest.

## Publisher’s note

All claims expressed in this article are solely those of the authors and do not necessarily represent those of their affiliated organizations, or those of the publisher, the editors and the reviewers. Any product that may be evaluated in this article, or claim that may be made by its manufacturer, is not guaranteed or endorsed by the publisher.
